# Antimicrobial peptides isolated from probiotics as an alternative to antibiotics against *Salmonella* infection

**DOI:** 10.1128/aem.01654-25

**Published:** 2026-01-30

**Authors:** Menuka Bhandari, Dhanashree Lokesh, Anusree Thenissery, Rajeev Shrestha, Gireesh Rajashekara

**Affiliations:** 1 Department of Animal Sciences, The Ohio State University, Wooster, Ohio, USA; 2 Department of Pathobiology, College of Veterinary Medicine, University of Illinois Urbana-Champaign 70154, Urbana, Illinois, USA; Anses, Maisons-Alfort Laboratory for Food Safety, Maisons-Alfort, France

**Keywords:** *Salmonella*, antimicrobial peptides, poultry, food safety

## Abstract

Non-typhoidal *Salmonella* (NTS) is the leading cause of deaths associated with foodborne illnesses in the United States. NTS is primarily transmitted in humans through the consumption of contaminated poultry and poultry products. Antibiotics and vaccines are used to control *Salmonella* infection in poultry. However, the evolution of antibiotic-resistant *Salmonella* and the lack of cross-protection by vaccines necessitate the development of novel antimicrobials for *Salmonella* infection. Antimicrobial peptides are considered viable alternatives due to the reduced propensity for resistance development, broad-spectrum activity, and lower toxicity. Our previous study detected 33 small peptides in the culture supernatants of *Lacticaseibacillus rhamnosus* GG and *Bifidobacterium lactis* (Bb12). Among them, we characterized the efficacy of two peptides (PN3 and PN5) against *Salmonella in vitro* and *in vivo*. Our results demonstrated that PN3 and PN5 inhibited the growth of *Salmonella* Typhimurium (ST) and nine other *Salmonella* serotypes. PN3 was cidal to ST within 30 min of incubation, and PN5 in 8 h in the time-kill kinetics assay. Additionally, both peptides completely cleared intracellular ST and ST inside the biofilm. PN3 and PN5 possessed stability at high temperatures and against proteolytic enzyme. In resistance assays, ST did not acquire resistance to both peptides. PN3 and PN5 were non-toxic to wax moths and increased their survivability and reduced ST load following challenge. Moreover, the oral administration of PN3 and PN5 significantly reduced ST load in the cecum of infected chickens. Overall, our study showed that PN3 and PN5 are promising candidates to control *Salmonella* infection in poultry.

**IMPORTANCE:**

Non-typhoidal *Salmonella* (NTS) is the leading cause of foodborne-associated deaths in the United States, with 420 deaths reported annually. Although antibiotics are used to control NTS invasive infection, overuse of antibiotics has accelerated the evolution of multidrug-resistant (MDR) *Salmonella*, necessitating the development of novel alternatives to antibiotics. Antimicrobial peptides (AMPs) are promising alternatives to antibiotics due to their activity against MDR *Salmonella*, lower chances of acquiring resistance, selectivity, and stability. This study investigated the effect of two AMPs, PN3 and PN5, against *Salmonella*. Our results demonstrated that PN3 and PN5 inhibit the growth of *Salmonella*, are stable at higher temperatures, and are resistant to proteolytic enzyme activity. *Salmonella* exposed to peptides were not prone to acquire resistance. Peptides were effective against *Salmonella* in the wax moth model and chickens. This study characterized two AMPs identified previously with potential for developing a novel approach to control *Salmonella* in poultry, prevent foodborne illnesses, and mitigate the rising antimicrobial resistance problem.

## INTRODUCTION

Non-typhoidal *Salmonella* (NTS) is the second leading cause of gastrointestinal infection in Europe (1) and foodborne-associated death in the United States (2). The US Centers for Disease Control and Prevention (CDC) has estimated approximately 1.35 million infections, 26,500 hospitalizations, and 420 deaths associated with salmonellosis in the United States annually and considers drug-resistant *Salmonella* as a serious public health threat (3, 4). The primary mode of *Salmonella* transmission in humans is through the consumption of contaminated poultry products such as eggs and meat (5). Human salmonellosis linked with eggs or egg-containing food consumption in the United States, Europe, and Australia is estimated at 29%, 20.5%, and 36%, respectively (6). Between 2015 and 2018, eggs contaminated with *Salmonella* Enteritidis in Poland caused the largest outbreak of salmonellosis in Europe (7). In 2013–2014, the CDC reported an outbreak of *Salmonella* Heidelberg from contaminated chicken products involving 634 people across 29 states in the United States and Puerto Rico (8).

Poultry can be infected with *Salmonella* vertically or horizontally. Vertical transmission occurs when *Salmonella* colonizes the reproductive tract of birds and is subsequently transmitted to the eggs (9). Horizontal transmission of *Salmonella* occurs from the environment, feed, vectors such as litter beetles, rodents, and other means. Infected birds regularly shed *Salmonella* with or, more commonly, without clinical signs. Studies have shown that healthy birds could be a reservoir of pathogenic and multidrug-resistant (MDR) *Salmonella* (10). Antibiotics such as enrofloxacin, tetracycline, and colistin sulfate were used to control *Salmonella* infection in poultry (11). However, the extensive use of antibiotics is associated with the emergence of antibiotic-resistant *Salmonella* (12). The transmission of antibiotic-resistant *Salmonella* strains, along with their resistance genes, from poultry to humans poses a significant threat to public health. In Canada, ceftiofur-resistant *Salmonella* strains isolated from humans were thought to have evolved on chicken farms that fed ceftiofur to treat *Salmonella* infections (13). Likewise, the use of antibiotics in poultry disturbs the gut microbiota, increases the abundance of opportunistic pathogens, enhances host susceptibility to infections, and slows the growth rate (14).

Vaccines are commonly used to protect poultry from *Salmonella* infection. Most of the commercial vaccines available in the market, such as Poulvac ST, Salmonella vac E, and Salmonella vac T, target only a few serotypes, such as *Salmonella* Typhimurium, *Salmonella* Enteritidis, or *Salmonella* belonging to serogroups B, C, and D (15, 16). Nevertheless, vaccines produced for specific serotypes do not provide cross-protection or only confer partial protection to diverse *Salmonella* serovars (17, 18). Additionally, live attenuated vaccine strains have been reported to persist in the commercial poultry production chain from production to processing and pose the risk of reverting to virulent strains (15). Therefore, there is an urgent need to develop novel antimicrobials that are less susceptible to acquiring bacterial resistance, possess broad-spectrum activity against multiple serovars of *Salmonella*, and are safe.

Antimicrobial peptides (AMPs) are promising alternatives to conventional antimicrobials to control bacterial infections due to their antimicrobial activity against broad-spectrum pathogens and MDR bacteria (4), immunomodulatory properties (19), low propensity to develop resistance (20), ability to potentiate the activity of other drugs (21), low toxicity, and high selectivity (20). In contrast to antibiotics, which target the specific biosynthetic pathway, AMPs interact with the negative charge of the bacterial cell membrane (20). The electrostatic interaction between the peptide’s positive charge and the negative charge of the bacterial cell wall, lipopolysaccharides in gram-negative bacteria, and teichoic acid in gram-positive bacteria leads to increased permeability, leakage of cellular contents, and lysis of bacterial cells (22). Several studies have isolated AMPs from a wide range of species: plants (*Medicago truncatula*-NCR247, NCR335) (23), reptiles (snake-C-BF [24], frog-Ctx-Ha [25]), mammalian cells (P3 [26]), and probiotics (*Escherichia coli* K12-Mcc25 [27]) that inhibit the growth of *Salmonella* (28). Furthermore, *in vivo* administration of AMPs such as MccJ25 (11), Ctx(Ile21)-Ha (29), and HJH-3 (30) reduced the load of *Salmonella* in the cecum and internal organs, including the spleen and liver of chickens. The beneficial effects of AMPs are attributed to the maintenance of gut homeostasis and intestinal integrity, promoting optimum nutrient absorption, and reducing the secretion of proinflammatory cytokines (30). Therefore, AMPs are promising candidates to control *Salmonella* infection in chickens.

This study extends our previous findings on the inhibitory effects of the PN peptides on *Salmonella* growth (31). In this comprehensive characterization, we evaluated the anti-*Salmonella* activity of novel AMPs, PN3 (VQAAQAGDTKPIEV) and PN5 (VTDTSGKAGTTKISNV), against the model strain *Salmonella* Typhimurium (ST). The biofilm eradication activity, ability to remove intracellular *Salmonella*, stability, and toxicity of PN3 and PN5 were tested *in vitro*. Furthermore, the *in vivo* efficacy of PN3 and PN5 against *Salmonella-*infected wax moths and chickens was investigated.

## RESULTS

### Physicochemical properties of PN3 and PN5

**Fig 1:**
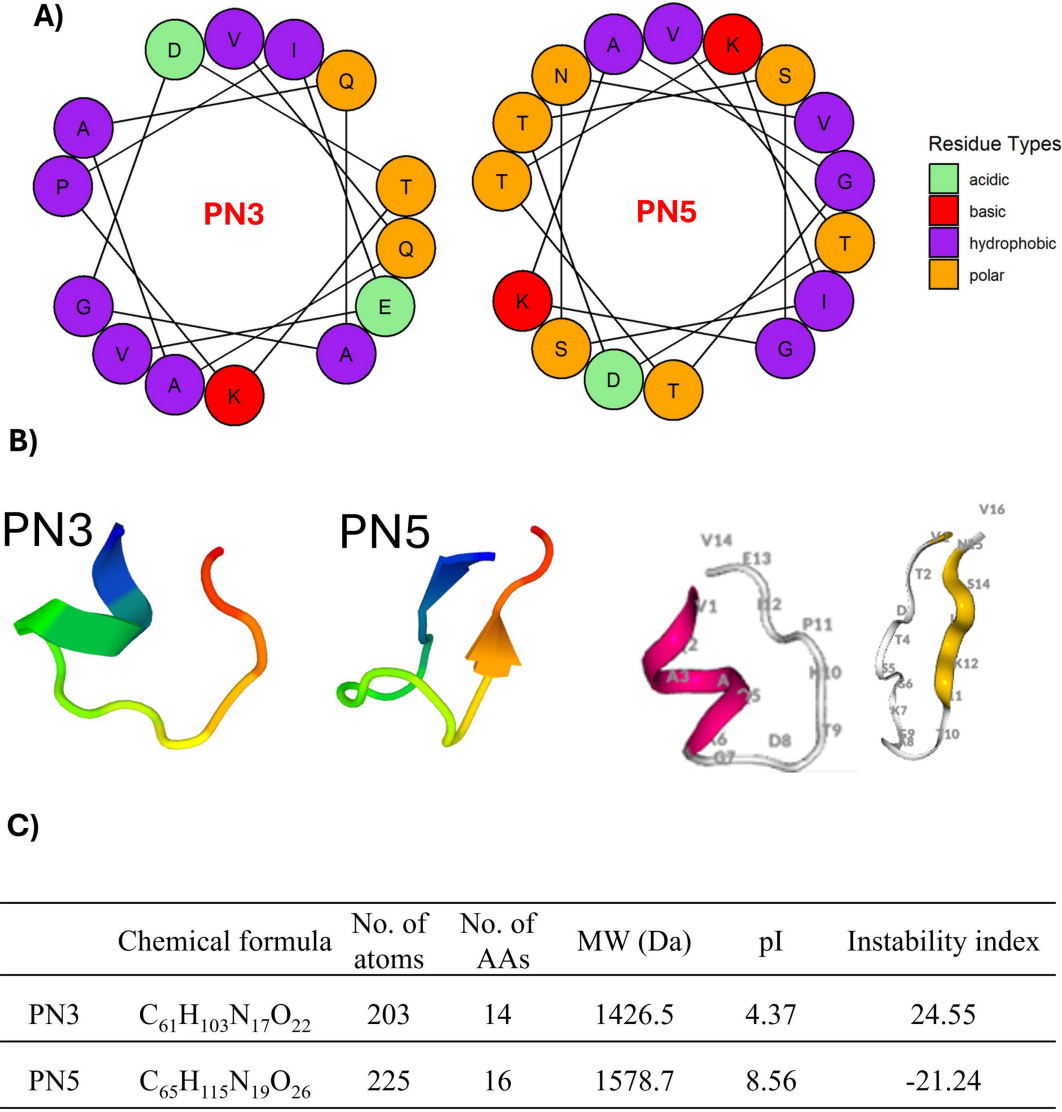
(**A**) Helical wheel diagram of PN3 and PN5 peptides showing the relative position of different AAs. (**B**) 3D models of PN3 and PN5 peptides predicted by PEP-FOLD, where the number represents the AA position. (**C**) Physical and chemical characteristics of PN3 and PN5 peptides: AAs = amino acids, MW = molecular wt, pI = isoelectric point.

The sequence of PN3 is “VQAAQAGDTKPIEV” and PN5 “VTDTSGKAGTTKISNV.” *In silico* analysis using the ProtParam tool predicted that both PN3 and PN5 are stable peptides, with an estimated half-life of 100 (mammalian reticulocytes, *in vitro*), >20 (yeast, *in vivo*), and >10 h (*E. coli*, *in vivo*). The predicted half-life of peptides in ProtParam is based on the N-end rule, which depends on the identity of the N-terminal amino acid (AA). The helical wheel projection of the peptide showed that hydrophobic and non-hydrophobic AAs were distributed as imperfect structures (Fig. 1A). The 3D structure of PN3 and PN5 predicted by PEP-FOLD showed that PN3 and PN5 consist of alpha helices and beta sheets, respectively (Fig. 1B). Additional physicochemical characteristics of PN3 and PN5 are provided in Fig. 1C. PN3 consists of 14 AAs with a predicted molecular mass of 1,426.5 Da, while PN5 contains 16 AAs with a predicted molecular mass of 1,578.7 Da. Both peptides are stable with the appropriate instability index below 40.

### PN3 and PN5 displayed broad-spectrum anti-*Salmonella* activity *in vitro*

**Fig 2:**
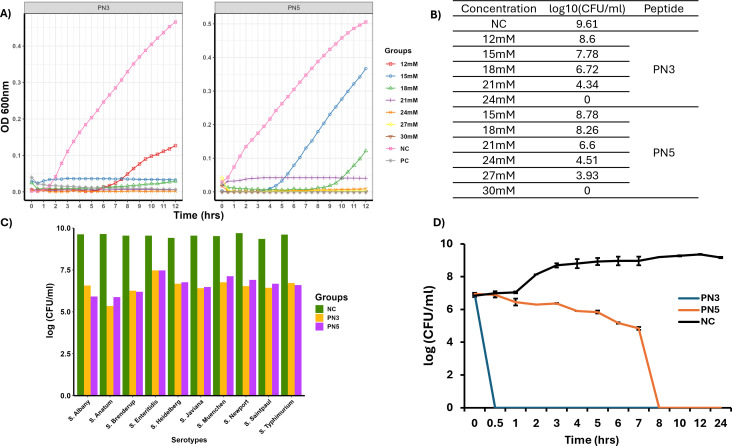
(**A**) MIC and MBC determination of PN3 and PN5. Different concentrations of peptides (12–30 mM) were added in a well containing 100 µL of 5 × 10^6^ CFU/mL ST and incubated at 37°C for 12 h. MIC is the minimum concentration that inhibits visible growth of ST, while MBC is the minimum concentration at which no viable bacteria are recovered by plating following incubation. (**B**) Log CFU of ST obtained after incubating ST with different concentrations of peptides for 12 h and plating in LB agar. (**C**) Broad-spectrum activity of PN3 and PN5. The inhibitory effect of the MIC of PN3 and PN5 peptides was tested against 10 serotypes of *Salmonella*. (**D**) Time-kill kinetics assay to assess the bactericidal effect of PN3 and PN5. ST was treated with MBC of peptides, and aliquots of bacteria were plated at different time points and expressed as log (CFU/mL). NC: Bacteria alone.

Thirty-three small peptides were detected in the supernatant of a mixture of *Lacticaseibacillus rhamnosus* GG (LGG) and Bb12 (32). Five highly abundant peptides that are common to LGG and Bb12 were tested for anti-*Salmonella* activity (32), and two peptides, PN3 and PN5, showed enhanced inhibition in *in vitro* conditions. The anti-*Salmonella* activity of the PN3 and PN5 peptides was initially evaluated against the model strain ST by measuring their minimum inhibitory concentration (MIC) and minimum bactericidal concentration (MBC) across a concentration range of 12 to 30 mM. Our data showed that the MIC and MBC of PN3 were 18 and 24 mM, whereas PN5 were 21 and 30 mM, respectively (Fig. 2A and B). Furthermore, MIC was used to check the broad-spectrum activity of peptides against nine different *Salmonella* serovars frequently reported in human illnesses. At the MIC, both peptides inhibited the growth of all nine *Salmonella* serovars as shown in Fig. 2C. Furthermore, a time-kill kinetics curve was generated to evaluate the time of onset of the bactericidal effect of PN3 and PN5 against ST using the MBC of peptides. As shown in Fig. 2D, both PN3 and PN5 killed ST in a time-dependent manner. Notably, PN3 had a rapid onset of bactericidal activity and killed ST within 30 min of incubation, while the untreated bacteria control had 6.99 ± 0.12 log CFU/mL at 30 min. PN5 gradually decreased the viability of ST, with complete killing observed at 8 h of incubation, whereas the untreated control group had 9.24 ± 0.03 log CFU/mL of ST at 8 h.

### PN3 and PN5 effectively eradicated biofilm-embedded *Salmonella*

The biofilm formation ability of ST on the minimum biofilm eradication concentration (MBEC) pegs was assessed by staining pegs with crystal violet (CV). Pegs containing ST retained a blue color after staining with CV. Consistent with it, the optical density (OD_600_) of the solution after dissolving CV-stained biofilm in glacial acetic acid was 0.74, while the media alone was 0.14. This result suggests that ST forms a biofilm (12).

The effect of PN3 and PN5 on the viability of biofilm-embedded ST was evaluated by incubating MBEC pegs with the preformed ST biofilm with the peptides, followed by plating. We observed that PN3 (18 mM), PN5 (21 mM), and reference control (kanamycin: 50 µg/mL) completely removed the biofilm-embedded ST, as no viable colonies were recovered after plating the culture (Table S1). In contrast, the untreated pegs had 7.6 log CFU/mL of ST in the biofilm (Table S1). Furthermore, a statistically significant difference in *Salmonella* load was observed in the PN3, PN5, and positive control (PC) groups compared to the negative control (NC) group (*P* < 0.05).

### PN3 and PN5 peptides cleared intracellular *Salmonella*

**Fig 3:**
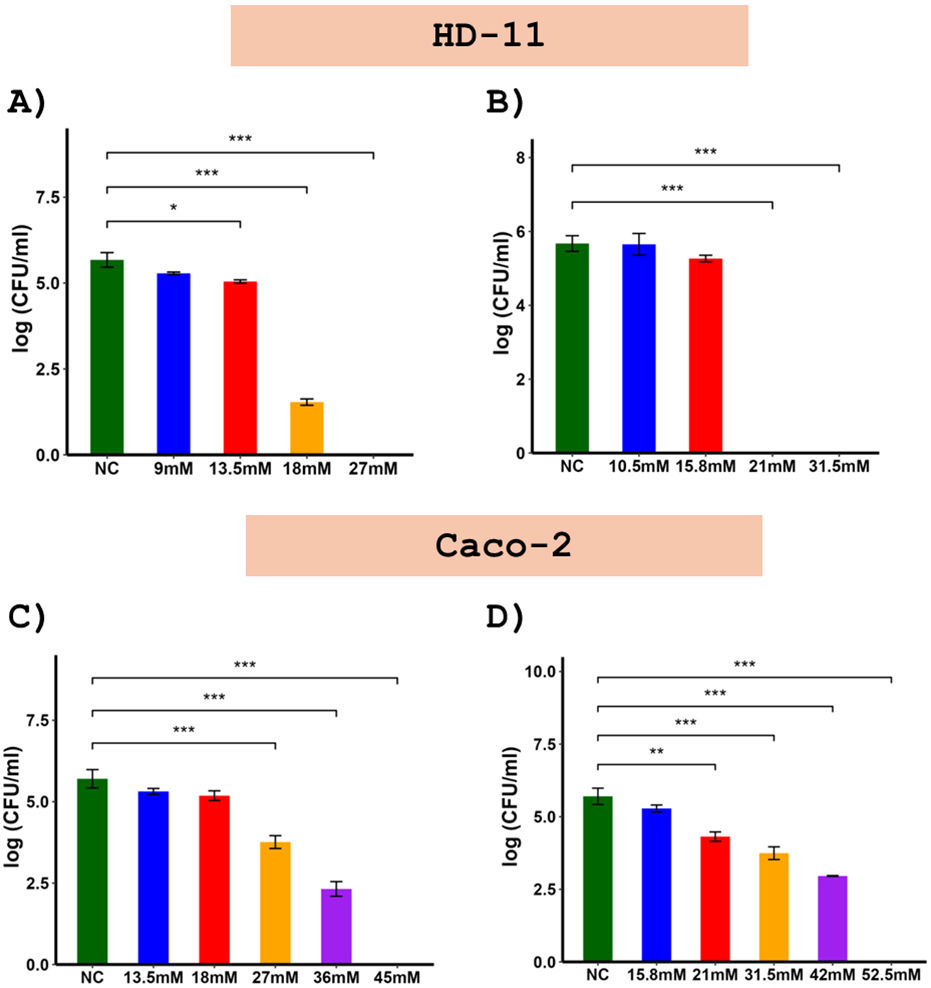
Efficacy of PN3 and PN5 to remove intracellular *Salmonella* from HD-11 (**A and B**) and Caco-2 (**C and D**) cells using gentamicin protection assay. Both cells were infected with ST at a multiplicity of infection of 100, incubated with peptides for 6 h, lysed with 0.1% Triton X-100, and plated to determine the intracellular ST. The data are presented as means ± standard deviation of results (*n* = 2).

The efficacy of PN3 and PN5 in clearing the intracellular *Salmonella* in HD-11 and Caco-2 cells was evaluated by using the gentamicin protection assay (Fig. 3A through D). Our results showed that the effect of PN3 and PN5 against the internalized *Salmonella* in both cells was concentration-dependent, with higher concentrations resulting in greater reduction of *Salmonella*. When the concentration of PN3 was 27 mM and PN5 was 21 mM, the complete eradication of the intracellular *Salmonella* was observed in HD-11 cells (Fig. 3A and B). However, HD-11 cells infected with *Salmonella* but not treated with peptide had 5.6 logs of *Salmonella*. Similarly, PN3 at 45 mM and PN5 at 52.5 mM completely killed *Salmonella* in Caco-2 cells (Fig. 3C and D). Caco-2 cells infected with *Salmonella* but not treated with peptide had 5.7 logs of *Salmonella*.

### PN3 and PN5 were stable in high temperatures and after PK treatment

**Fig 4:**
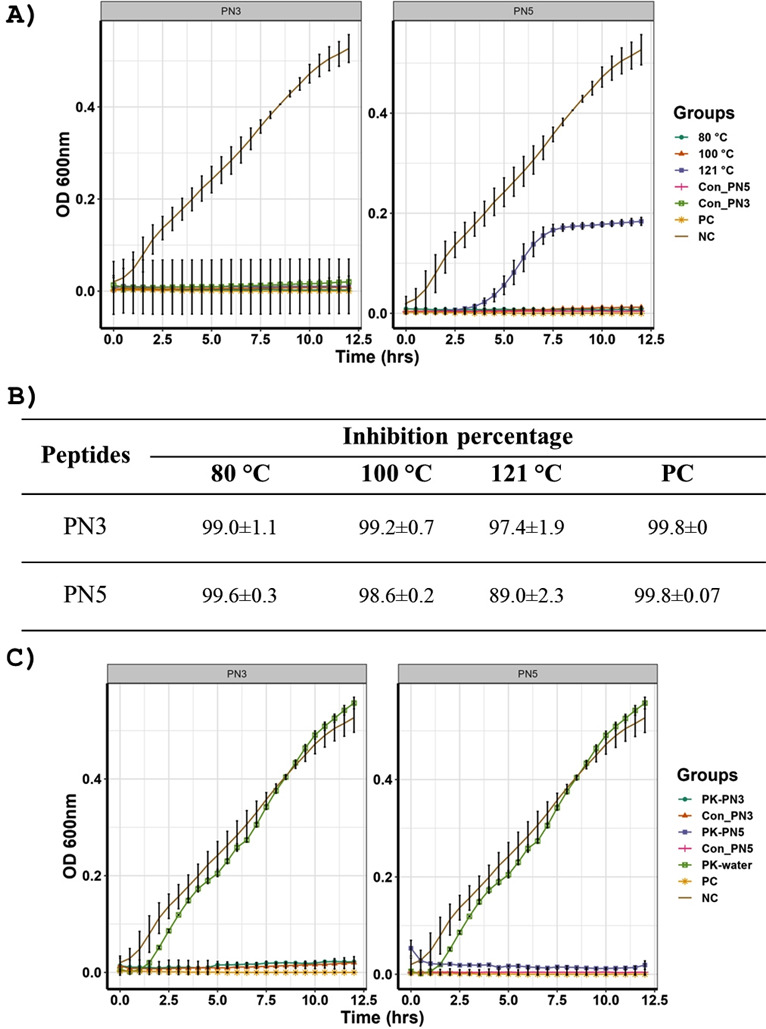
(**A**) Thermal stability of PN3 and PN5. Thermal stability was tested by incubating peptide at 80°C for 1 h, 100°C for 30 min, and 121°C for 20 min, followed by assessing MIC using micro-broth dilution. (**B**) The percentage inhibition of ST by PN3 and PN5 after exposure in different temperatures. (**C**) PK stability of PN3 and PN5. Proteinase stability was determined by incubating peptides with 1 mg/mL of PK for 2 h at 37°C, followed by assessing MIC using micro-broth dilution. Con_PN3: non-treated PN3, Con_PN5: non-treated PN5, PK-PN3: PN3 incubated with PK, PK-PN5: PN5 incubated with PK, PK-water: bacteria treated with PK. PC: positive control bacteria treated with kanamycin (50 ug/ml), NC: negative control bacteria not treated

The thermal stability of PN3 and PN5 was investigated by incubating the peptides at 80°C, 100°C, and 121°C and measuring the inhibitory activity. Figure 4A shows that the activity of both PN3 and PN5 did not change after incubating peptides at 80°C and 100°C. Similarly, the activity of PN3 was not affected after incubating the peptide at 121°C (Fig. 4A). However, the incubation of PN5 at 121°C slightly reduced its activity (Fig. 4A). Nevertheless, the plating of ST treated with PN5 incubated at 121°C showed that the inhibition was 89% (Fig. 4B). Both PN3 and PN5 retained stability after treatment with the proteinase K (PK) (Fig. 4C). The activity of PN3 and PN5 was similar before and after the treatment with PK. Hence, PN3 and PN5 possess thermal and proteolytic enzyme stability.

### *Salmonella* did not acquire resistance to PN3 and PN5

**Fig 5:**
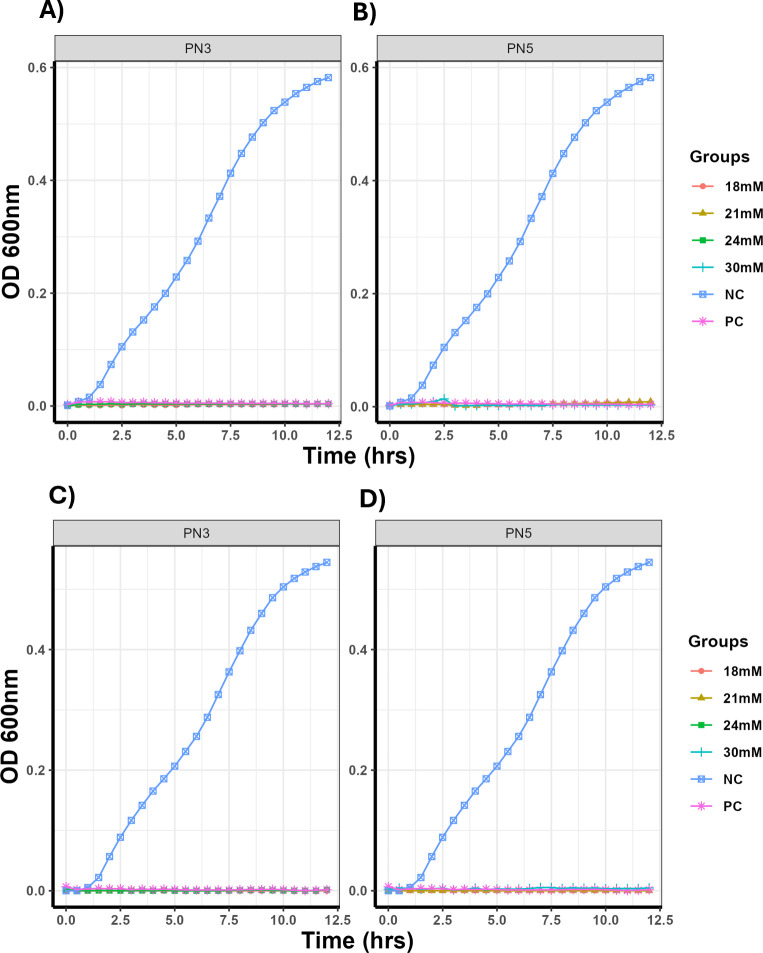
Growth curve of ST obtained from the sub-lethal (**A and B**) and lethal (**C and D**) resistance assays. For the sub-lethal resistance assay, ST was repeatedly passed in the sub-inhibitory concentration of peptides 13 times. After the 13th passage, MIC and MBC of the culture were determined. For lethal resistance assay, *Salmonella* was treated with 2× MBC of peptides for 2 days, and the colonies grown on the agar plates were tested for MIC and MBC.

The ability of *Salmonella* to gain resistance to PN3 and PN5 was evaluated by the sub-lethal and lethal resistance assays. In the sub-lethal resistance assay, ST was sub-cultured in Luria Bertani (LB) broth containing a sub-inhibitory concentration of peptide (0.75× MIC), and MIC and MBC were tested after the 13th passage. The results revealed that repeated exposure of ST to the peptide did not change the MIC and MBC of the peptide (Fig. 5A and B). Similarly, in lethal resistance assay, *Salmonella* was treated with 2× MBC of peptides for 2 days, after which the surviving colonies were tested for MIC and MBC. The results showed no significant change in inhibitory activity against *Salmonella* (Fig. 5C and D).

### Antagonism of PN3 with PN5

Combined therapy is commonly practiced to potentiate the activity of drugs. In this study, PN3 and PN5 were combined at various concentrations to determine the combination index (CI). The plating data indicated a higher bacterial count when PN3 and PN5 were combined at concentrations of 18 and 21 mM, compared to PN3 alone (Fig. S1). The CI was computed using the concentration of PN3 and PN5 that inhibited the growth of ST. Our results demonstrated that the CI of PN3 and PN5 peptides was 1.85, suggesting an antagonistic effect on ST. Similar to our findings, apidaecin has been reported to exhibit antagonistic interactions with pexiganan (33). The antagonistic activity among peptides could be due to competition for binding sites (34), which can reduce their ability to reach the target at the required concentration (34, 35).

### PN3 and PN5 were non-toxic to wax moth larvae and reduced the *Salmonella* load in wax moth larvae

**Fig 6:**
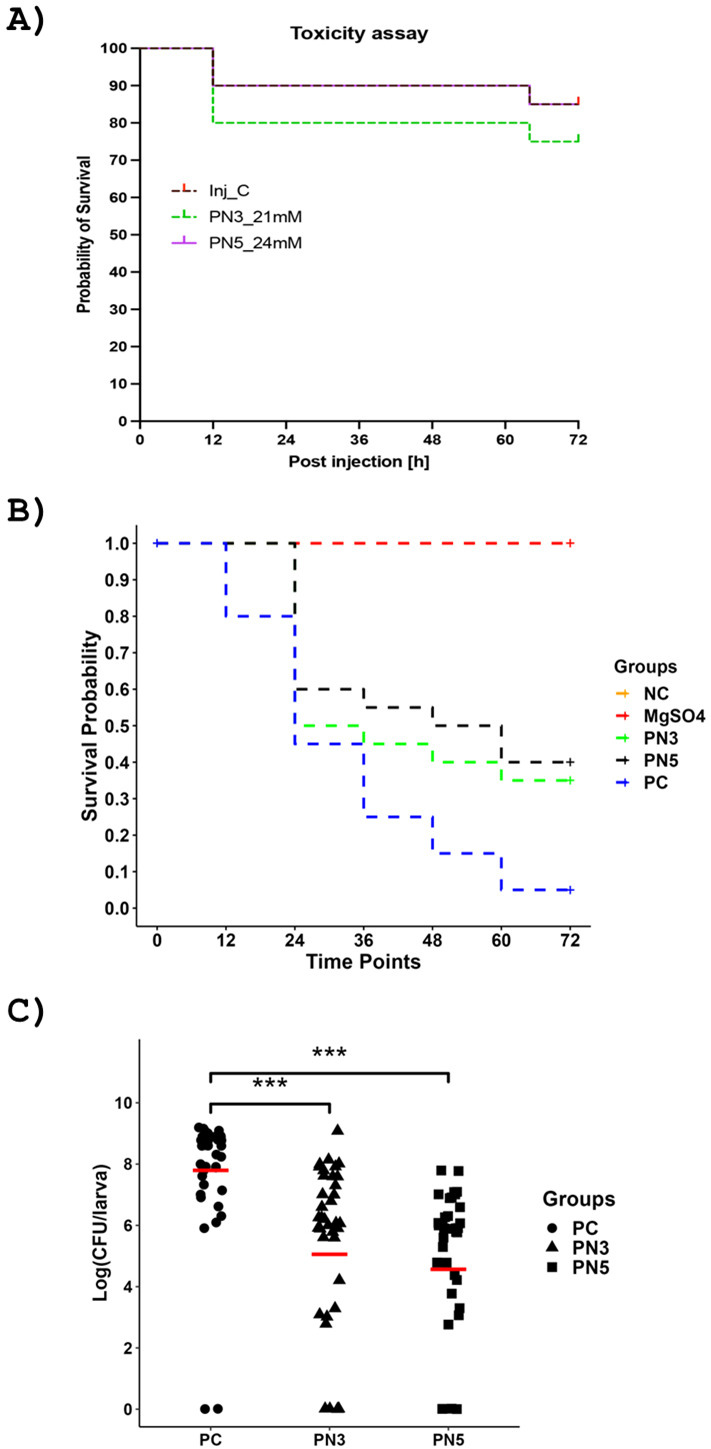
*In vivo* toxicity and efficacy of PN3 and PN5 against ST-infected wax moth (*Galleria mellonella*) larvae. (**A**) Kaplan-Meier survival curves depicting the toxicity of PN3 and PN5 peptides in wax moth larva (*n* = 20/group). PN3 (21 mM) and PN5 (24 mM) were injected into wax moth larvae, and survivability was monitored every 12 h for 3 days. (**B**) Kaplan-Meier survival curves of ST-infected larvae treated with PN3 (18 mM) and PN5 (21 mM). Larvae (*n* = 40) were injected with peptides 2 h before the infection with 10^4^ CFU/larva of ST. Survivability of larvae was monitored in a similar way to toxicity for 3 days. (**C**) Quantification of ST load in wax moth larvae. For survivability and ST load quantification, *n* = 40 larvae/group were used. NC: non-infected and non-treated, Inj_C/MgSO*4*: MgSO_4_ injected, PC: ST-infected, PN3_21 mM: PN3-injected (21 mM), PN5_24 mM: PN5-injected (24 mM), PN3: ST-infected and PN3-injected (18 mM), PN5: ST-infected and PN5-injected (21 mM).

*In vivo* toxicity and efficacy of PN3 and PN5 were assessed in the wax moth larvae. To determine the toxicity, PN3 (21 mM) and PN5 (24 mM) were injected through the last prolegs of wax moth larvae, and larvae were monitored for 72 h. The mortality of the larvae was monitored as a peptide toxicity endpoint, along with melanization and behavioral changes (larval mobility). Before larva death, the larva changes its color from golden to dark black and becomes immobile even after touching with pipette tips. There will be dark larvae that will die, or the golden/cream color larvae (live) at the termination of the experiment. Our results showed that the injection of the wax moth larvae with PN3 and PN5 had no statistically significant effect on larval survival rate compared to the magnesium sulfate (MgSO_4_)-injected control group (Fig. 6A).

The efficacy of the PN3 and PN5 against *Salmonella-*infected wax moth larvae was evaluated by injecting the larvae with the peptides 2 h before the infection with ST. Survivability of larvae was monitored for 72 h, and the load of ST was determined. Our results demonstrated that 95% of larvae infected with *Salmonella* (PC) were dead by 72 h (Fig. 6B). However, the mortality of larvae treated with PN3 (18 mM) and PN5 (21 mM) decreased significantly compared to the *Salmonella-*infected group. The survivability of PN3 (18 mM) and PN5 (21 mM)-treated larvae after 72 h was 35% and 40%, respectively (*P* < 0.05) (Fig. 6B). Furthermore, the load of ST in the larvae was significantly reduced in PN3- and PN5-treated groups compared to the untreated group. The average log reduction of ST in the PN3 group was 2.35, and the PN5 group was 2.45, respectively, compared to the untreated group (Fig. 6C).

### PN3 and PN5 reduced the *Salmonella* load in the cecum of chickens

**Fig 7:**
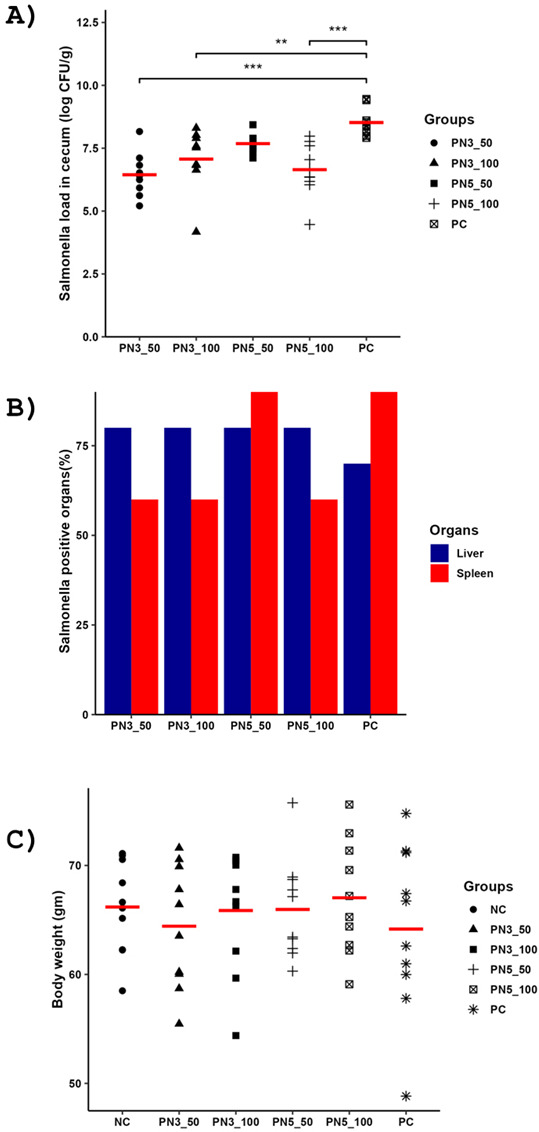
*In vivo* efficacy of PN3 and PN5 against the ST-infected chickens. PN3 (50 and 100 mg/kg) and PN5 (50 and 100 mg/kg) were administered twice a day orally from day 1 to day 5. On day 2, birds were orally infected with 4 × 10^4^ CFU of ST and necropsied on day 7. (**A**) ST load in the cecum on 5 dpi. (**B**) Percentage of birds positive for ST in liver and spleen on 5 dpi. (**C**) Body weight of chickens infected with ST and treated with peptides on 5 dpi, *N* = 10/group. NC: non-infected and non-treated, PN3_50: infected with ST and treated with PN3 (50 mg/kg), PN3_100: infected with ST and treated with PN3 (100 mg/kg), PN5_50: infected with ST and treated with PN5 (50 mg/kg), PN5_100: infected with ST and treated with PN5 (100 mg/kg), PC: ST-infected.

To determine the efficacy of PN3 and PN5 against ST-infected chickens, peptides were administered orally at two doses (50 and 100 mg/kg) twice daily for 5 days. We selected 50 and 100 mg/kg because our earlier study found that P1 and P2 peptides at these doses significantly reduced avian pathogenic *E. coli* (APEC) load in the cecum of chickens (36). Our results showed that PN3 at 50 and 100 mg/kg and PN5 at 100 mg/kg significantly reduced the load of ST in the cecum (*P* < 0.05) (Fig. 7A). Similarly, the PN3 at 50 mg/kg, PN3 at 100 mg/kg, and PN5 at 100 mg/kg groups demonstrated a 30% reduction in chickens positive for ST in the spleen compared to the PC (not treated infected with *Salmonella*) group (Fig. 7B). However, none of the treatment groups reduced the ST in the liver. Further, the PC group had the lowest body weight compared to all other groups on 5 days post-infection (dpi). However, the changes in body weight were not statistically significant between the groups (*P* > 0.05) (Fig. 7C).

### Alanine scanning defined AA residues important for the activity of PN3 and PN5 peptides

**Fig 8:**
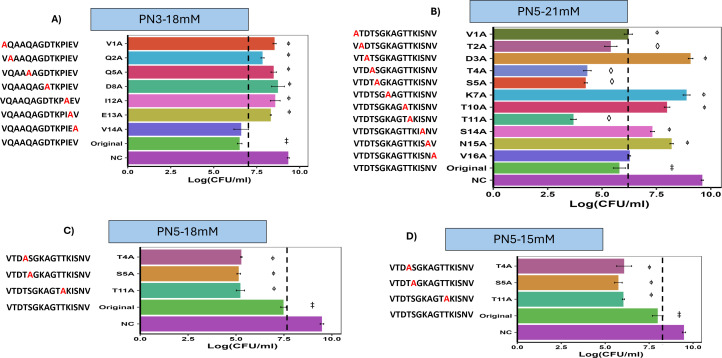
Alanine scanning to identify the AA residue required for the activity of the PN3 and PN5. The AA residue of the original peptide was replaced with alanine. (**A and B**) Bacterial viability of ST after 12 h incubation with either the original peptide (PN3 and PN5) or their alanine-substituted analogs, presented as log-transformed CFU counts. The PN3 and its analogs were treated at 18 mM, whereas PN5 and its analogs were treated at 21 mM. (**C and D**) Dose response analysis of three PN5-alanine-substituted analogs (T4A, S5A, and T11A), tested at 18 and 15 mM. Different symbols indicate statistically significant differences in the log bacterial counts of ST after treatment with peptide analogs, compared to the original peptide. “**ϕ”** represents significant increase (*P* < 0.05) in the log bacterial counts of ST after treatment with peptide analogs, compared to the original peptide. **“◊”** represents significant reduction (*P* < 0.05) in the log bacterial counts of ST after treatment with peptide analogs, compared to the original peptide. (*P* < 0.05). NC: bacteria alone, and original: non-substituted peptide.

Alanine scanning was conducted to determine the AA residues important for the bioactivity of peptides. Positively charged AAs, lysine (K) and arginine (R), are important for the electrostatic interaction of AMPs with bacterial membranes (37). Hydrophobic AAs, phenylalanine, isoleucine (I), and valine (V) are crucial for the insertion of the peptide into the bacterial membrane, permeabilization, and destabilization of the bacterial membrane (38). In PN3, each AA was replaced by alanine except glycine (G) as its substitution reduces the structural stability of the peptide (39). In addition to the G residue, the I was not replaced in PN5. A total of 10 analogs of PN3 and 12 analogs of PN5 were commercially synthesized. Two analogs of PN3 (K10A: VQAAQAGDTAPIEV, P11A: VQAAQAGDTKAIEV) and one analog of PN5 (K12A-VTDTSGKAGTTAISNV) were not included due to the solubility issue in water. One analog of PN3 (T9A: VQAAQAGDAKPIEV) precipitated during the incubation with ST at 37°C. Our results showed that six alanine-substituted analogs of PN3 (V1A, Q2A, Q5A, D8A, I12A, and E13A) significantly reduced the ST inhibitory activity compared to the original peptide (Fig. 8A). Similarly, six alanine-substituted analogs of PN5 (V1A, D3A, K7A, T10A, S14A, and N15A) demonstrated significant reduction in inhibitory activity compared to the original peptide (Fig. 8B).

Through alanine scanning, we found three analogs of the PN5 peptide (T4A, S5A, and T11A) that exhibited significant improvement in activity compared to the non-substituted original PN5 at 21 mM (Fig. 8B). Furthermore, we evaluated the MIC of these three PN5 analogs in 18 and 15 mM against ST using broth microdilution method. The result demonstrated that both concentrations of the analogs significantly reduced bacterial growth compared to the native PN5, with an MIC of 15 mM (Fig. 8C and D). At 15 mM, the T4A, S5A, and T11A analogs demonstrated additional log reductions of 1.91 (*P* < 0.005), 2.23 (*P* < 0.005), and 1.95 (*P* < 0.005), respectively, in *Salmonella* viability compared to the original peptide. Similarly, at 18 mM, these analogs exhibited reductions of 2.20 (*P* < 0.0005), 2.33 (*P* < 0.00005), and 2.25 (*P* < 0.0005) log units, respectively, relative to the original peptide.

The hydrophobicity of the peptide analogs T4A (−0.175), S5A (−0.169), and T11A (−0.175) was greater than that of the parent peptide PN5 (−0.331). Additionally, other analogs also demonstrated improved hydrophobicity. However, no significant changes in antimicrobial activity were observed for the other analogs. Other parameters, such as stability and helical wheel projections, were not changed compared to the parent peptide. The helical wheel projections and physicochemical properties of the peptide analogs are shown in Fig. S2.

### Increasing the net charge by R substitution did not change the MIC of PN3 and PN5 peptides

The higher net positive charge of the AMPs is associated with higher antimicrobial activity due to the increased electrostatic interaction between the AMPs and the negatively charged phospholipid of bacteria (37). Earlier studies have shown that the substitution of positively charged AAs such as K and R yielded significant improvement in the antimicrobial activity of peptides (37). To examine the effect of increased net charge on the activity of PN3 and PN5 peptides, negatively charged AAs such as aspartic acid (D) and glutamic acid (E) of the original peptide sequence were replaced by R (Table S2). Three R-substituted analogs of PN3 (D8R: VQAAQAGRTKPIEV; E13R: VQAAQAGDTKPIRV; and D8R and E13R: VQAAQAGRTKPIRV) and one analog of PN5 (D3R: VTRTSGKAGTTKISNV) were assessed for the inhibitory property against ST using the broth microdilution method. The PN3 analogs were tested at 15 and 18 mM, while the PN5 analog was tested at 18 and 21 mM. Our results demonstrated that in both concentrations, the analogs of single substitution of PN3 (D8R and E13R) showed similar activity as the original peptide, whereas substituting with two R (D8R and E13R) in the same peptide decreased the activity compared to the original peptide (Fig. S3A and B). Similarly, the R-substituted analog of PN5 (D3R) demonstrated similar activity as original peptide (Fig. S3C and D).

## DISCUSSION

AMPs are promising agents to control infectious pathogens due to their potent antimicrobial activity against MDR infections (4, 40), a broad spectrum of anti-bacterial activity (4, 41), anti-biofilm activity (42), and lower chances of resistance development (21). AMPs isolated from probiotic bacteria have emerged as a promising alternative to conventional antibiotics, particularly in combating MDR pathogens such as *Salmonella* (28). In this study, two peptides, PN3 and PN5, isolated from LGG and Bb12 were further studied for their anti-*Salmonella* activities. *In silico* physicochemical analysis showed that both peptides are stable, with favorable half-lives and instability indices below 40. Both PN3 and PN5 exhibit alpha-helical and beta-sheet conformation and imperfect amphipathic distributions favorable for membrane interaction. Our results demonstrated that both PN3 and PN5 exhibited broad-spectrum anti-*Salmonella* activity against 10 different serovars of *Salmonella* associated with foodborne illnesses. Previous studies indicated that AMPs derived from probiotics displayed anti-*Salmonella* activity (27). Furthermore, consistent with our study, Festa et al. (42) reported inhibitory activity of 1018-K6 peptide against different serovars of *Salmonella*. Indeed, our results showed that PN3 and PN5 are bacteriostatic at lower concentrations and bactericidal at higher concentrations. At MBC, PN3 killed *Salmonella* within 30 min of incubation, and PN5 killed *Salmonella* at 8 h. Consistent with our results, Wu et al. (43) demonstrated the dose-dependent effect of the AMPs against the MDR enterotoxigenic *E. coli*. The ability of PN3 and PN5 to inhibit the growth of different *Salmonella* serovars suggests the potential use of PN3 and PN5 to control *Salmonella* infection.

Similar to the growth inhibition, we also found that the MIC of PN3 and PN5 can completely remove biofilm-embedded *Salmonella*. There are several stages of biofilm formation in *Salmonella*: initial attachment/adhesion of bacteria, upregulation of quorum sensing genes, and interaction between the extracellular polymeric substance (EPS) (44). Biofilm formation is one of the key mechanisms by which *Salmonella* becomes resistant to antimicrobials and causes chronic infections (45). Therefore, there is a necessity to develop novel antimicrobials that are effective against both the biofilm and planktonic *Salmonella*. Some AMPs can prevent the formation of biofilm or alter the structure of biofilm by reducing the thickness of biofilm, while others can eradicate bacteria inside the biofilm by damaging the membrane (46). In our study, the MIC of peptides completely removed the *Salmonella* embedded in the biofilm. Similar to our study, Mataraci et al. (47) showed that the MIC of peptide eradicated biofilm-embedded *Staphylococcus aureus*. Likewise, Nisin at MIC almost completely inhibited the biofilm formation and reduced the viable cells inside the biofilm in *Streptococcus suis* (46). The bactericidal property of PN3 and PN5 against biofilm-embedded *Salmonella* could be due to several factors, such as the physiological state of bacteria, permeability of the bacterial membrane, and enhanced interaction of peptides with EPS, which needs further exploration (48).

The toxicity and stability are major factors that limit the commercialization of AMPs. In our study, PN3 (21 mM) and PN5 (24 mM), above their MIC, did not demonstrate toxicity to wax moth larvae. There was no significant difference in the survivability of wax moth larvae in the peptide-injected group compared to the control group. Likewise, stability at higher temperatures is an essential property required for AMPs that are intended to be used in poultry feed because the feeds are generally pelleted at 83°C (49). In our study, both PN3 and PN5 were thermostable up to 100°C and exhibited no change in MIC after incubating peptides at 100°C for 30 min. Consistent with our findings, earlier studies have shown that peptides isolated from probiotics were stable at 90°C (50) and 121°C (51). However, the activity of PN5 was slightly reduced after heating at 121°C for 15 min. Similarly, the activity of the peptides produced by *Bacillus* was decreased when incubated at 121°C (52). Small peptides under 10 kDa are generally less susceptible to thermal degradation (53). Molecular weights of PN3 and PN5 are 1.4 and 1.5 kDa, respectively, which might have prevented them from thermal degradation. Further, the natural rearrangement of the AAs can mask the cleavage site for proteolytic enzymes, thus leading to likely enhanced proteolytic stability of peptides (54). The thermal stability of PN3 and PN5 suggests that both PN3 and PN5 can be incorporated into the poultry feed for delivery, a common practice in the poultry industry.

*Salmonella* enters the host cell either through phagocytic mechanisms using immune cells or the non-phagocytic mechanism using epithelial cells (55). After entering the host cell, being an intracellular pathogen, *Salmonella* resides inside the immune cells such as macrophages (56). Macrophages help in the invasion as well as systemic dissemination of *Salmonella* (55). Therefore, the intracellular bactericidal activity of PN3 and PN5 was assessed in the Caco-2 (epithelial cell) and HD-11 (macrophage cell). The PN3 and PN5 completely removed the intracellular *Salmonella* in both the Caco-2 and HD-11 cells (Fig. 3A through D), implying that these peptides can reduce the invasion and systemic spread of *Salmonella*. Similarly, our chicken experiment results showed that administering PN3 and PN5 reduced the percentage of birds positive for *Salmonella* in the spleen, suggesting the reduced systemic dissemination of *Salmonella* in peptide-treated group. The different concentrations of peptides required to remove *Salmonella* from HD-11 and Caco-2 cells might be due to the differences in the cell type and the level of subcellular accumulation of the peptides in the phagolysosomes (57). Macrophages are reported to engulf pathogens and microparticles, which might have caused the internalization of more peptides compared to the epithelial cell line (57).

The wax moth larval infection model and chicken infection model were used to further verify the *in vivo* efficacy of the peptides. PN3 and PN5 increased the survival rate of wax moth larvae infected with ST (Fig. 6B). Similar to our findings, P1 and P2 peptides reduced the load of APEC in wax moth larvae (36), and CecA peptide isolated from insects improved the survivability of wax moth larvae infected with uropathogenic *E. coli* (58). Additionally, a significantly lower load of *Salmonella* was observed in the wax moth injected with PN3 and PN5 peptides (Fig. 6C). Similar to the wax moth data, the oral administration of PN3 at 50 and 100 mg/kg and PN5 at 100 mg/kg to chickens significantly reduced the load of ST in the cecum and spleen. In our study, both peptides significantly reduced *Salmonella* load in the cecum and, to a lesser extent, in the spleen, but not in the liver. This may be attributed to differences in metabolic stability of the peptides between the liver and spleen. Previous studies have shown that peptidase expression and activity are higher in the liver compared to the spleen (59, 60), suggesting that peptides may be metabolized in the liver, thereby reducing their efficacy in the liver. Consistent with our findings, the administration of HJH-3 peptides to chickens and mice significantly reduced the load of *Salmonella* in the spleen (30, 61). Supplementation of MccJ25 for 8 days reduced the *Salmonella* infection rate compared to the untreated challenge group (11). The authors attributed this effect to modulation of the gut microbiota, reduced secretion of proinflammatory cytokines, and enhanced intestinal integrity (11). Administration of Ctx(Ile21)-Ha for 28 days reduced the mortality rate in chickens infected with *Salmonella* (29). Therefore, PN3 and PN5 are promising candidates for preharvest control of *Salmonella* infection.

In summary, PN3 and PN5 possess broad-spectrum anti-*Salmonella* activity against diverse *Salmonella* serovars of food safety significance. They are stable at high temperatures, resistant to PK, and induce no resistance. PN3 and PN5 are effective against biofilm-embedded *Salmonella*, intracellular *Salmonella*, and chickens. Our results lay a foundation for the development of a novel antimicrobial against salmonellosis. However, further studies on understanding the mechanisms of action and evaluating the efficacy of PN3 and PN5 by supplementing the drinking water of chickens are needed to exploit the translational potential of these peptides to control *Salmonella* infection in poultry and consequently reduce the incidence of human salmonellosis.

Our study has some limitations. Although we observed a significant improvement in the survivability of the larva groups treated with the peptides, there were still some mortalities observed, which could be attributed to a lower dose of the peptides used in this study (62). Previous studies have shown the dose-dependent effect of the antimicrobials in wax moth (62, 63), with higher doses resulting in greater reduction in pathogen load. Thus, using a higher dose might provide better protection. Therefore, further dose optimization studies are needed to enhance the effect of peptides. The choice of doses for PN3 and PN5 was based on our prior work involving P1 and P2 against APEC (36) and *Salmonella* (64), where similar doses demonstrated efficacy. Additionally, the selected doses are within the range of commonly used antibiotics to control salmonellosis in clinical settings (65). Although PN3 and PN5 are distinct peptides from the P1 and P2 peptides, the current study was conducted as a pilot proof-of-concept evaluation of peptides for their potential effect *in vivo*. Therefore, we decided to use PN3 and PN5 at doses similar to those used for P1 and P2 in our earlier study. In the present study, although the peptide-treated groups demonstrated numerically higher body weights compared to the PC, the differences were not statistically significant. We acknowledge that other performance metrics, such as feed conversion ratio, could provide additional insights into the impact of the peptides.

## MATERIALS AND METHODS

### Bacterial strains and growth conditions

The details of the strains used in this study and the growth conditions are provided in Table S3. ST was used as a model strain to characterize the activity of peptides. Different serotypes of *Salmonella*, commonly associated with foodborne illness, were used to determine the broad-spectrum activity of peptides. Nalidixic acid-resistant (Nal^r^) *Salmonella* was used for wax moth and chicken experiments. All strains were aerobically grown in LB broth or LB plate and incubated at 37°C for 12–24 h, otherwise stated.

### Synthesis of peptides and *in silico* sequence analysis

PN3 and PN5 are short-chain peptides that were detected in the supernatant of LGG and Bb12 (32). For all the *in vitro* and *in vivo* assays, PN3 and PN5 were commercially synthesized from GenScript (NJ, USA) with purity of >95%, and dissolved in water. The physicochemical properties of PN3 and PN5 were predicted using the HeliQUest server. The isoelectric point (pI), liposolubility index, average hydrophobic index, and instability index were calculated using the ProtParam tool (https://web.expasy.org/). Protein with an instability index value less than 40 is considered stable, whereas more than 40 is considered unstable (https://web.expasy.org/). The helical wheel projection was determined using the “helixvis” package in R.4.3.1 studio. The tertiary structure of the peptide was predicted using the PEP-FOLD3 server.

### MIC and MBC determination

The MIC and MBC of the PN3 and PN5 peptides were determined by the broth microdilution method as previously described (36). ST was grown overnight in LB broth, and OD_600_ was adjusted to 0.05 (~5 × 10^6^ CFU/mL). An aliquot of 100 µL of adjusted culture was added to a sterile, non-treated, flat-bottom 96-well plate (Corning Inc., Corning, NY, USA), followed by the addition of various concentrations of peptides ranging from 12 to 30 mM. The plate was incubated in a Tecan Sunrise kinetic microplate reader (Tecan Group Ltd. Switzerland) at 37°C for 12 h. The OD_600_ was measured every 30 min with shaking between each measurement. MIC is the lowest concentration of peptide that completely inhibits the visible growth of *Salmonella*. The inhibition percentage was calculated [(OD_600_ NC – OD_600_ peptide)/OD_600_ NC] × 100. Cultures without visible growth were directly plated on LB agar to determine the MBC. The agar plate was incubated at 37°C for 24 h. Controls included were bacteria alone (NC), bacteria with 50 µg/mL kanamycin (PC), and media control (sterility).

### Activity against different serovars of *Salmonella*

MICs of the peptides determined above were tested against nine additional *Salmonella* serovars by following the above protocol (36). *Salmonella* Albany, *Salmonella* Anatum, *Salmonella* Braenderup, *Salmonella* Enteritidis, *Salmonella* Javiana, *Salmonella* Heidelberg, *Salmonella* Muenchen, *Salmonella* Newport, and *Salmonella* Saintpaul were tested to evaluate the broad-spectrum activity of PN3 and PN5.

### Time-kill kinetics

Time-kill assay was conducted according to the previously described protocol (46). ST was grown overnight at 37°C and sub-cultured for 3 h. The OD_600_ of the log phase culture was adjusted to 0.05 (~5 × 10^6^ CFU/mL), exposed to MBC of PN3 (24 mM) and PN5 (30 mM) in an Eppendorf tube, and incubated at 37°C by shaking at 200 rpm. To determine the viability of culture, 10 μL aliquots were taken from each sample at 0, 0.5, 1, 2, 3, 4, 5, 6, 7, 8, 10, 12, and 24 h, serially diluted, and plated on LB agar. Plates were incubated at 37°C for 24 h. Bacteria alone (NC) and media alone were used as controls. The mean log_10_ CFU/mL of each peptide at different times was plotted in the kinetic kill curve.

### Biofilm formation by CV staining and MBEC assay

CV staining method was performed to test the ability of ST to form biofilm on MBEC (Innovotech, Edmonton, AB, Canada) pegs. The efficacy of the peptide against biofilm-embedded ST was assessed by the MBEC assay (66). To determine the biofilm formation ability, an aliquot of 180 µL of ST grown in LB media was adjusted to 0.05 OD_600_ (~5 × 10^6^ CFU/mL) and added into wells of the MBEC device containing polystyrene pegs. The MBEC plate was incubated at 37°C for 36 h. Pegs were washed with phosphate-buffered saline (PBS) to remove loosely adherent planktonic bacteria. To visualize the biofilm formed on pegs, pegs were stained with 200 µL of 0.4% CV, dried for 20 min, and the stain was dissolved in 33% glacial acetic acid (12). An absorbance microplate reader was used to measure the OD of the ST at 600 nm. The biofilm formation of the ST-treated pegs was compared with the control well incubated with just the media to classify ST as a low, moderate, or high biofilm producer.

The bactericidal effect of PN3 and PN5 was evaluated by the MBEC assay (high throughput). ST biofilm was formed in the MBEC pegs as described above. After biofilm formation, loosely attached bacteria were removed by washing with PBS. The biofilm-formed pegs were transferred to a new plate containing peptides at their MIC and incubated at 37°C for 18 h. After incubation, pegs were transferred to a new 96-well plate containing 1× PBS and sonicated for 30 min (Aquasonic ultrasonic cleaner, VWR) to disrupt the biofilm. The sonicated culture was 10-fold serially diluted, plated on LB agar, and incubated at 37°C for 24 h to determine the viable colonies. Bacteria alone (NC), kanamycin (50 µg/mL: PC), and media were used as controls. The experiment was conducted two times.

### Activity against the intracellular *Salmonella* in HD-11 and Caco-2 cells

The gentamicin protection assay was performed to check the efficacy of peptides in clearing the intracellular ST from HD-11 and Caco-2 cells (67). Cells were plated in a 96-well plate (10^5^ cells/well), and the media were replaced with the incomplete media, without fetal bovine serum and antibiotics, 2 h before infection. The overnight-grown ST was sub-cultured in LB media, and cells were infected with ST at a multiplicity of infection of 100. HD-11 cells were incubated for 1 h, whereas Caco-2 was incubated for 3 h post-infection. The cells were washed and treated with gentamicin (150 μg/mL) to remove extracellular ST. Cells were treated with different concentrations of peptides for 6 h, lysed with 0.1% Triton X-100, and plated on LB agar plate after a 10-fold serial dilution. Cells non-infected with ST and infected with ST but not treated with peptides were used as controls. The experiment was conducted two times.

### Effect of temperature on the activity of peptides

The purpose of treating peptides at higher temperatures is to mimic the feed processing steps in the feed industry. Our future aim is to use these peptides in poultry feed to control *Salmonella*. As poultry feeds are pelleted at very high temperatures, knowing the thermal stability of peptides is an important step before adding them to feed. Both PN3 and PN5 were treated at three temperatures for different times as specified: 80°C for 1 h, 100°C for 30 min, and 121°C for 20 min (68, 69). Peptides kept at room temperature, bacteria alone (NC), and kanamycin (50 µg/mL: PC) were used as controls. MIC of the heat-treated and untreated peptides was determined as described above. The experiment was conducted two times.

### Proteolytic stability of peptides

To examine proteolytic stability, both peptides were treated with PK (1 mg/mL) and incubated at 37°C for 2 h. The reaction was inactivated by incubating at 100°C for 10 min. MIC of PK-treated peptides was determined as described above. Untreated peptides, bacteria alone (NC), kanamycin (50 µg/mL: PC), and bacteria with PK (PK-water) were used as controls. The experiment was conducted two times.

### Resistance studies

The sub-lethal and lethal resistance assays were performed to check whether ST would acquire resistance to the peptides. Sub-lethal resistance assay was performed following the previously described protocol (36). Peptides at a sub-inhibitory concentration (0.75× MIC) were added in an Eppendorf tube with 100 µL of OD_600_ 0.05 (~5 × 10^6^ CFU/mL) of ST and incubated at 37°C. Then, 20 µL of overnight culture was mixed with 80 µL of the fresh LB media, followed by the addition of 0.75× MIC of peptides. This procedure was repeated 12 more times. After the 13th passage, MIC and MBC were determined as described above. Bacteria alone (NC), kanamycin (50 µg/mL: PC), and media alone were used as controls for both experiments.

For lethal resistance assay, ST (~10^10^ CFU/mL) was prepared from the overnight culture grown in LB media, 10 µL was mixed with the agar containing 2× MBC of peptides, and incubated for 2 days at 37°C (36). The ST grown on a plate with peptides was resuspended in 100 µL of LB media, transferred to a tube containing 5 ml of LB media, and incubated at 37°C for 12 h. MIC and MBC were determined as described above.

### Combination effect of PN3 and PN5

The combination effect of the PN3 and PN5 peptides was evaluated by following the combination assay as described previously (70, 71). For the combination activity, overnight-grown ST was adjusted to OD_600_ of 0.05 (~5 × 10^6^ CFU/mL), and 100 µL was added to a 96-well plate. Each peptide at different concentrations (7.5, 9, and 10.5 mM) was added to each well. MIC was determined as described above. Bacteria alone (NC) was used as a control. The CI was obtained using the following equation:


$$\;CI=\frac{C_{isoA}}{C_{A}}+\frac{C_{isoB}}{C_{B}}$$

where CI = combination index, *C*_isoA_= concentration of peptide A in combination, CA = concentration of peptide A individually, *C*_isoB_ = concentration of peptide B in combination, *C*_*B*_ = concentration of peptide B individually. CI value < 1, synergism; CI value > 1, antagonism; CI value = 1, additive.

### Wax moth (*Galleria mellonella*) toxicity and efficacy

The *in vivo* toxicity and efficacy of peptides were determined in wax moth as described previously (36). Wax moth is an ideal model to determine the *in vivo* efficacy of antimicrobials due to similar immune responses as mammals, lower cost, and replicable results (72). Upon receipt, the fifth instar wax moth larva was incubated in the dark at 37°C for 12 h. The cream-colored wax moth was selected for the study. For the toxicity determination, wax moths (*n* = 20 per group) were divided into four groups: non-injected control, magnesium sulfate-injected control (Inj_c), PN3-injected group, and PN5-injected group. PN3 (21 mM) and PN5 (24 mM) were injected through the last proleg of the wax moth using a PB600-1 repeating dispenser (Hamilton, Reno, NV, USA) attached to a 100 μL insulin syringe, 31-gauge 8 mm needle length (ReliOn, Bentonville, AR, USA). All larvae were incubated at 37°C in a petri dish, and mortality was monitored every 12 h for 72 h.

To determine efficacy, PN3 (18 mM) and PN5 (21 mM), MIC of peptides, were injected first in the last proleg 2 h before infection with 10^4^ CFU/larva of Nal^r^-resistant ST. The overnight-grown Nal^r^ ST was injected into another proleg. Larva was monitored in a similar way to toxicity for 72 h. Every 12 h post-infection, the dead larva (black-colored larva with no movement) was collected, macerated, dissolved in 1 mL 1× PBS, 10-fold serially diluted, and plated on the XLT-4 plate with Nal (50 µg/mL). Likewise, after 72 h, all the live larvae were collected, processed, and plated to determine CFU. The experiment was conducted two times.

### Chicken experiment

The *in vivo* efficacy of PN3 and PN5 peptides was further evaluated in chickens infected with *Salmonella*. Sixty specific pathogen-free layers (*n* = 10/group) were divided into six groups: NC (no ST infection and without treatment), PC (ST infection but no treatment), PN3_50 (ST-infected and treated with 50 mg/kg of PN3), PN3_100 (ST-infected and treated with 100 mg/kg of PN3), PN5_50 (ST-infected and treated with 50 mg/kg of PN5), and PN5_100 (ST-infected and treated with 100 mg/kg of PN5). The efficacy of PN3 and PN5 in reducing ST load in the cecum and internal organs was measured by administering peptides via oral gavage as described previously (36, 66). Two different doses of the PN3 and PN5 peptides (50 and 100 mg/kg) were given from day 1 to day 5 twice a day. The purpose of starting treatment a day earlier than the challenge is to simulate prevention and have enough peptide bioavailable in the chicken circulation. Chickens were orally challenged with Nal^r^ ST (4 × 10^4^ CFU) on day 2. On day 7, chickens were necropsied, and the cecum, liver, and spleen were processed in a similar way as described previously (66). Cecal suspension was serially diluted, plated on XLT-4 agar supplemented with 50 µg/mL of Nal, and incubated at 37°C for 24 h. The liver and spleen were plated directly as well as after enrichment. For enrichment, 1 mL of the homogenized liver and spleen suspension was added to the 9 mL of tetrathionate broth, incubated for 24 h at 37°C, and plated in XLT-4 agar containing Nal.

### Alanine scanning

To determine the key AA residues responsible for the antimicrobial activity of the peptide, the AA residue in the wild-type peptides was replaced with alanine (73). Alanine is a neutral AA with a short side chain. The single substitution of wild-type residue with alanine does not disrupt the secondary structure of the peptides (74). The inhibitory activity of each alanine-substituted peptide analog against ST was initially determined by testing the MIC of the peptides. Additionally, three PN5 analogs showing lower bacterial counts compared to the original peptide at MIC were further tested at 18 and 15 mM to determine the MIC. After 12 h, ST culture was serially diluted and plated on LB agar to quantify the bacteria in log (CFU/mL). Bacteria alone (NC) and the original peptide (non-substituted) were used as controls. The experiment was repeated twice.

### Substitution of the negatively charged AA residue of PN3 and PN5 with R

To increase the net charge of the PN3 and PN5 peptides, peptide analogs were commercially synthesized from GenScript (NJ, USA) (Table S2). In peptide derivatives, each D and E AA residue of the original peptides was substituted by R. The inhibitory activity of each R-substituted peptide analog against ST was determined by testing the MIC of the peptide analogs as described above. Bacteria alone (NC) and the original peptide (non-substituted) were used as controls.

### Statistical analysis

All data analysis was done in R 4.3.1. For the MBEC, intracellular survival assay, load of *Salmonella* in wax moths and cecum of chickens, and alanine scanning assay, one-way analysis of variance followed by Tukey’s *post hoc* test was used to compute the statistically significant differences between the treatment groups. Statistical difference in the survivability of wax moth larvae was calculated using the Kaplan-Meier log-rank test. *P*-value < 0.05 was considered statistically significant.

## SUPPLEMENTAL MATERIAL

Figure S1 aem.01654-25-s0001.tif Antagonism between PN3 and PN5 against *S.* Typhimurium.

Figure S2 aem.01654-25-s0002.pdf 3D model of the PN3 and PN5 alanine- and arginine-substituted peptide analogs.

Figure S3 aem.01654-25-s0003.tif Growth curve showing the antimicrobial activity of arginine-substituted analogs.

Supplemental legends aem.01654-25-s0004.docx Descriptive captions for supplemental material.

Supplemental tables aem.01654-25-s0005.docx Tables S1 to S3.

ASM does not own the copyrights to Supplemental Material that may be linked to, or accessed through, an article. The authors have granted ASM a non-exclusive, world-wide license to publish the Supplemental Material files. Please contact the corresponding author directly for reuse.
