# Supplementary figures and images for: Antimicrobial peptides isolated from probiotics as an alternative to antibiotics against Salmonella infection
Source: Appl Environ Microbiol. 2026 Jan 30;92(2):e01654-25. doi: 10.1128/aem.01654-25 (PMC12915304; doi:10.1128/aem.01654-25)

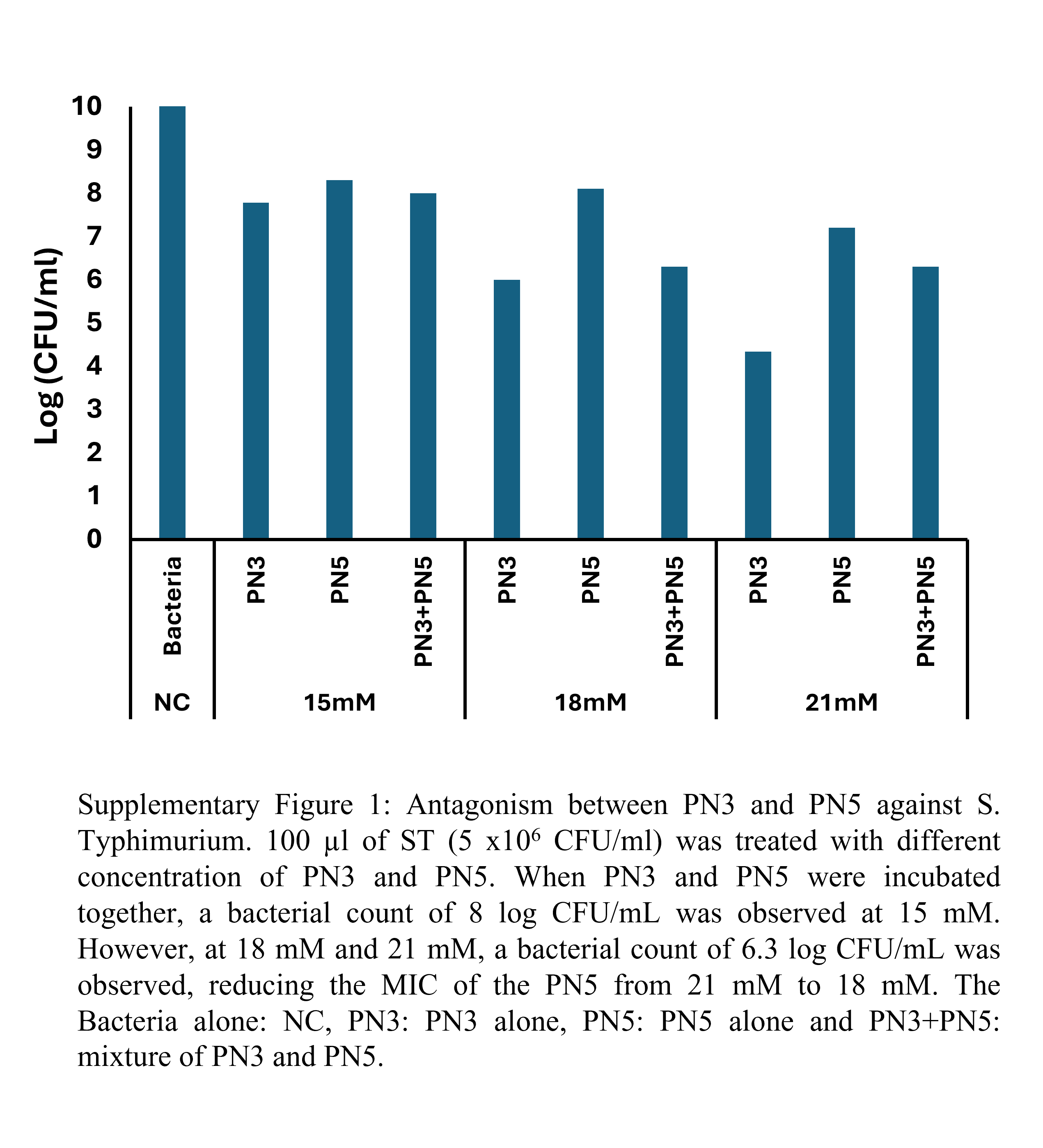

Supplement: Figure S1 — Antagonism between PN3 and PN5 against S. Typhimurium. [file aem.01654-25-s0001.tif]

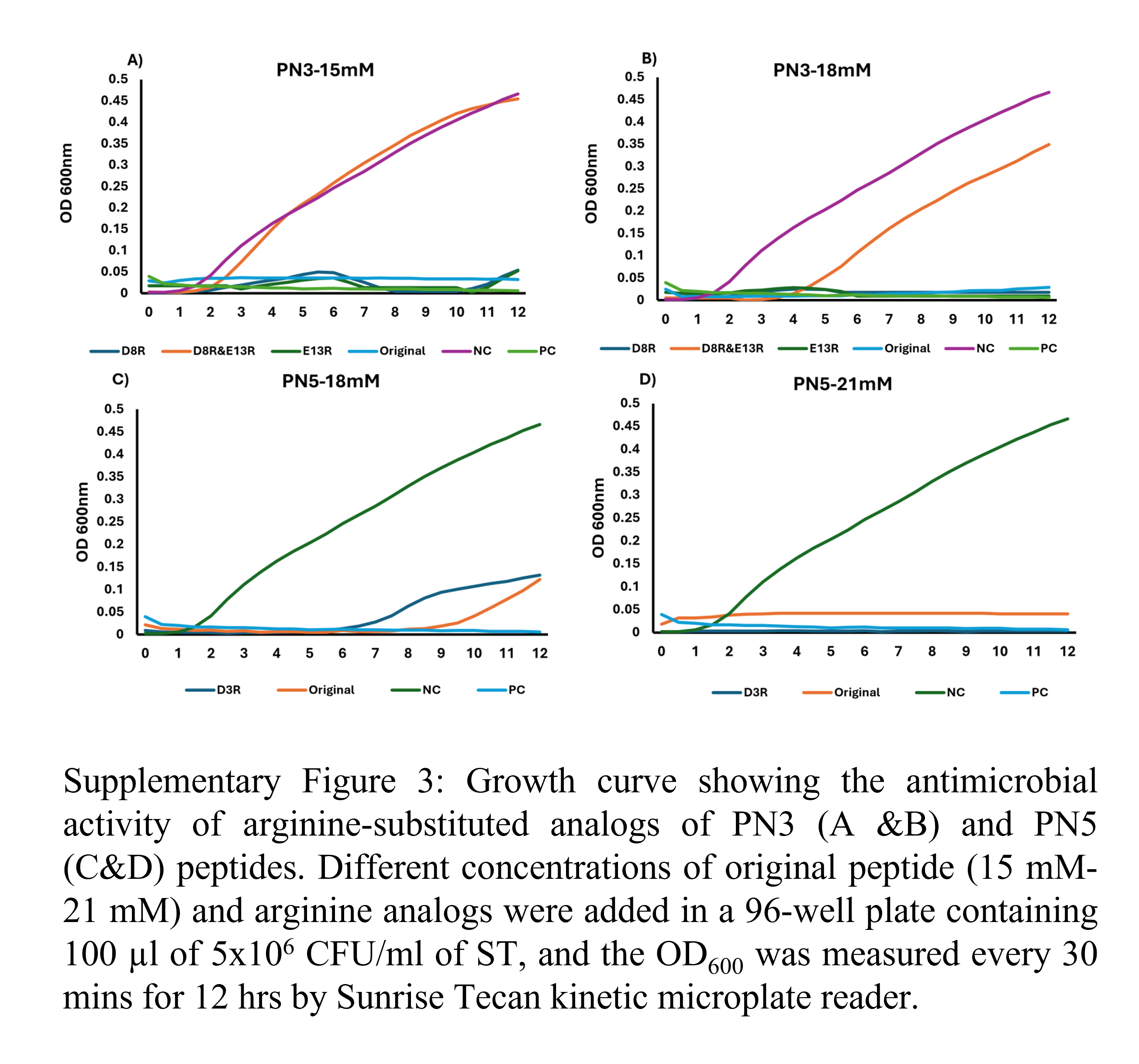

Supplement: Figure S3 — Growth curve showing the antimicrobial activity of arginine-substituted analogs. [file aem.01654-25-s0003.tif]
