# Supplementary material for: Antimicrobial peptides isolated from probiotics as an alternative to antibiotics against Salmonella infection
Source: Appl Environ Microbiol. 2026 Jan 30;92(2):e01654-25. doi: 10.1128/aem.01654-25 (PMC12915304; doi:10.1128/aem.01654-25)
Supplement: Figure S2 — 3D model of the PN3 and PN5 alanine- and arginine-substituted peptide analogs. [file aem.01654-25-s0002.pdf]

Supplementary Figure 2: **A and B** - 3D model of the PN3 and PN5 alanine and arginine substituted peptides analogs predicted by PEP-FOLD. **C and D** - Physiochemical properties of PN3 and PN5 alanine and arginine substituted peptide analogs, where AA=Amino acids, MW=Molecular weight, PI= Isoelectric point and GRAVY = Grand average of hydropathicity. **E and F** - Helical wheel diagram of PN3 and PN5 alanine and arginine substituted peptide analogs showing the relative position of different amino acids.

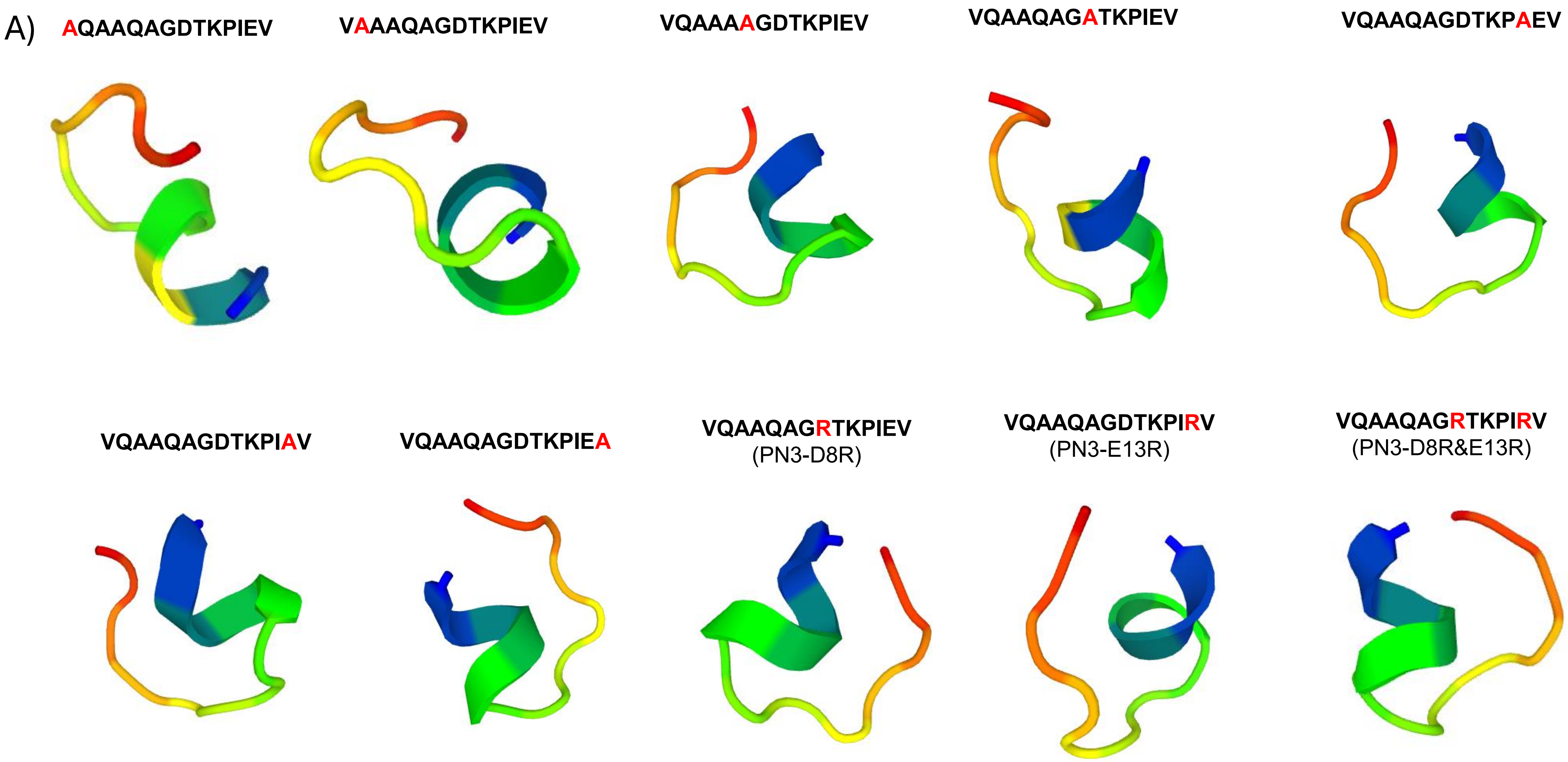

3D model of the PN3-alanine and arginine substituted peptides predicted by PEP-FOLD

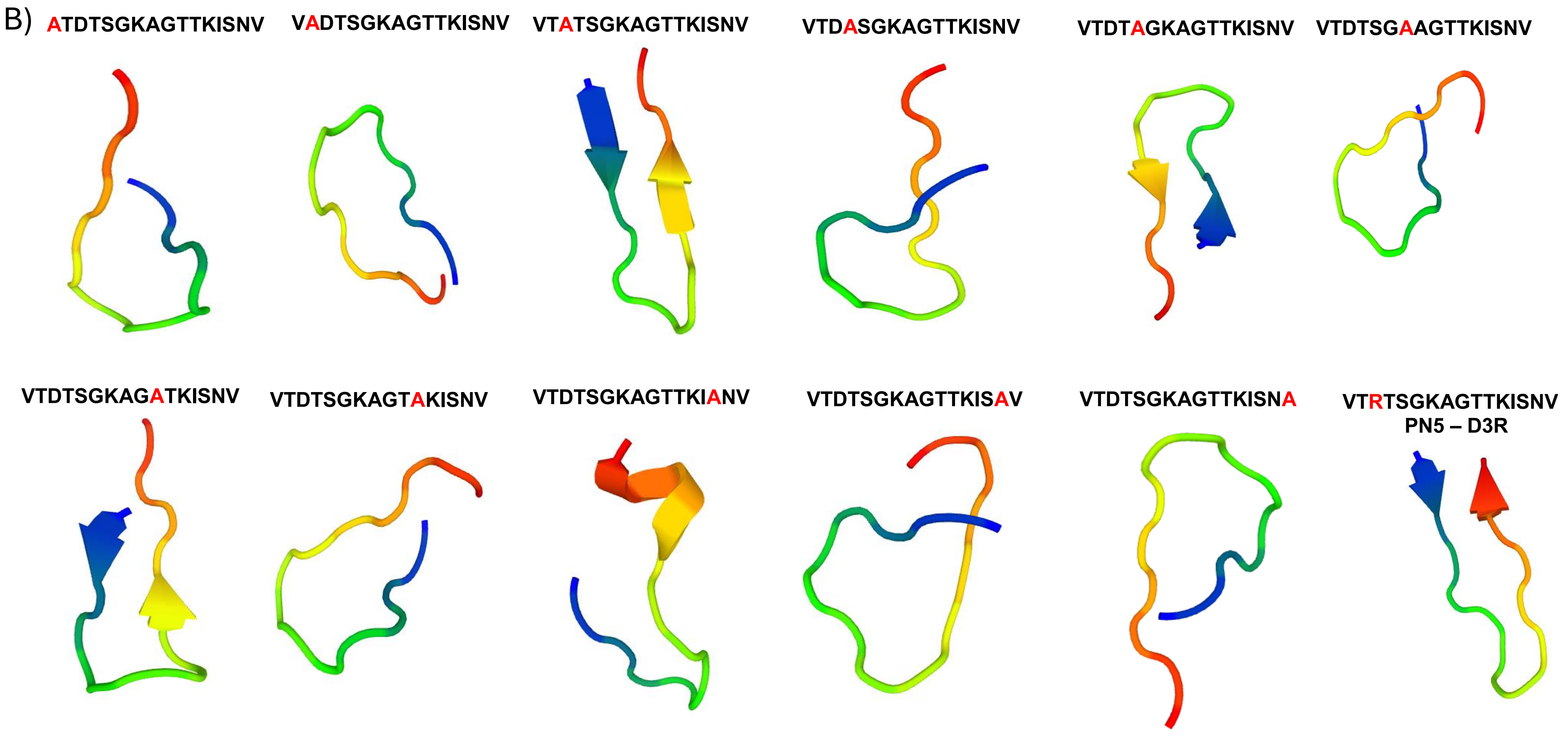

3D model of the PN5-alanine and arginine substituted peptides predicted by PEP-FOLD

C)

Peptides	Formula	# of atoms	# of AA	MW (Da)	PI	Instability index	GRAVY
AQAAQAGDTKPIEV	$C_{59}H_{99}N_{17}O_{22}$	197	14	1398.54	4.37	24.55	-0.336
VAAAQAGDTKPIEV	$C_{59}H_{100}N_{16}O_{21}$	196	14	1369.54	4.37	24.55	0.214
VQAAAAGDTKPIEV	$C_{59}H_{100}N_{16}O_{21}$	196	14	1369.54	4.37	24.55	0.214
VQAAQAGATKPIEV	$C_{60}H_{103}N_{17}O_{20}$	200	14	1382.58	5.97	29.22	0.214
VQAAQAGDTKPAEV	$C_{58}H_{97}N_{17}O_{22}$	194	14	1384.51	4.37	6.92	-0.357
VQAAQAGDTKPIAV	$C_{59}H_{101}N_{17}O_{20}$	197	14	1368.55	5.81	-6.84	0.214
VQAAQAGDTKPIEA	$C_{59}H_{99}N_{17}O_{22}$	197	14	1398.54	4.37	24.55	-0.336
VQAAQAGDTKPIEV	$C_{61}H_{103}N_{17}O_{22}$	203	14	1426.59	4.37	24.55	-0.164
VQAAQAGRTKPIEV (PN3-D8R)	$C_{63}H_{110}N_{20}O_{20}$	213	14	1467.69	8.72	35.29	-0.236
VQAAQAGDTKPIRV (PN3-E13R)	$C_{62}H_{108}N_{20}O_{20}$	210	14	1453.66	8.72	-6.84	-0.236
VQAAQAGRTKPIRV (PN3-D8R&E13R)	$C_{64}H_{115}N_{23}O_{18}$	220	14	1494.76	12.01	3.90	-0.307

Physiochemical properties of PN3-alanine and arginine substituted peptide analogs, where AA=Amino acids, MW=Molecular weight, PI= Isoelectric point and GRAVY = Grand average of hydropathicity

D)

Peptides	Formula	# of atoms	# of AA	MW (Da)	PI	Instability index	GRAVY
ATDTSGKAGTTKISNV	$C_{63}H_{111}N_{19}O_{26}$	219	16	1550.69	8.64	-15.94	-0.481
VADTSGKAGTTKISNV	$C_{64}H_{113}N_{19}O_{25}$	221	16	1548.71	8.56	-21.24	-0.175
VTATSGKAGTTKISNV	$C_{64}H_{115}N_{19}O_{24}$	222	16	1534.73	10.00	-11.85	0.000
VTDASGKAGTTKISNV	$C_{64}H_{113}N_{19}O_{25}$	221	16	1548.71	8.56	-11.85	-0.175
VTDTAGKAGTTKISNV	$C_{65}H_{115}N_{19}O_{25}$	224	16	1562.74	8.56	-21.24	-0.169
VTDTSGAAGTTKISNV	$C_{62}H_{108}N_{18}O_{26}$	214	16	1521.64	5.81	-21.24	0.025
VTDTSGKAGATKISNV	$C_{64}H_{113}N_{19}O_{25}$	221	16	1548.71	8.56	-21.24	-0.175
VTDTSGKAGTAKISNV	$C_{64}H_{113}N_{19}O_{25}$	221	16	1548.71	8.56	-21.24	-0.175
VTDTSGKAGTTKIANV	$C_{65}H_{115}N_{19}O_{25}$	224	16	1562.74	8.56	-21.24	-0.169
VTDTSGKAGTTKISAV	$C_{64}H_{114}N_{18}O_{25}$	221	16	1535.71	8.56	-21.24	0.000
VTDTSGKAGTTKISNA	$C_{63}H_{111}N_{19}O_{26}$	219	16	1550.69	8.56	-21.24	-0.481
VTRTSGKAGTTKISNV (PN5-D3R)	$C_{67}H_{122}N_{22}O_{24}$	235	16	1619.84	11.17	-11.85	-0.394

Physiochemical properties of PN5-alanine and arginine substituted peptide analogs, where AA=Amino acids, MW=Molecular weight, PI= Isoelectric point and GRAVY = Grand average of hydropathicity

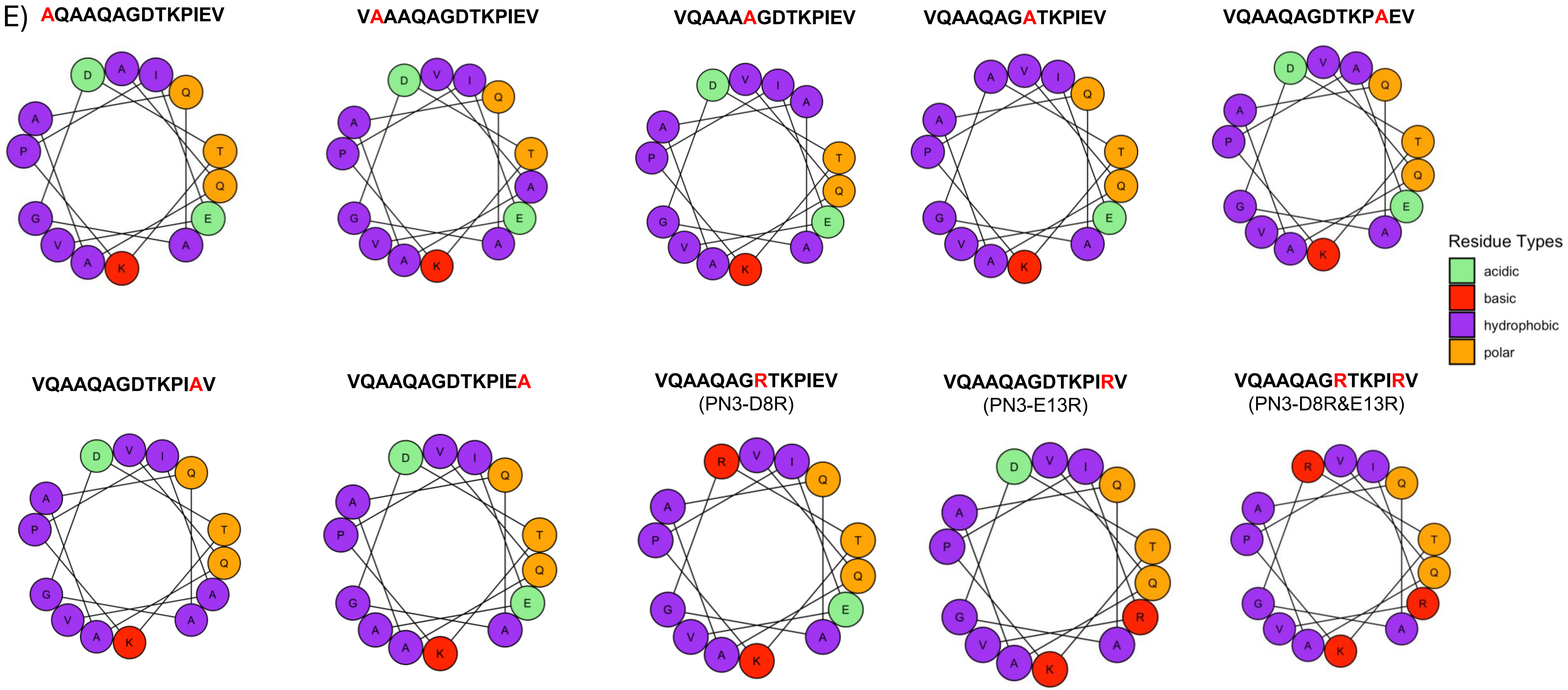

Helical wheel diagram of PN3-alanine and arginine substituted peptide analogs showing the relative position of different amino acids.

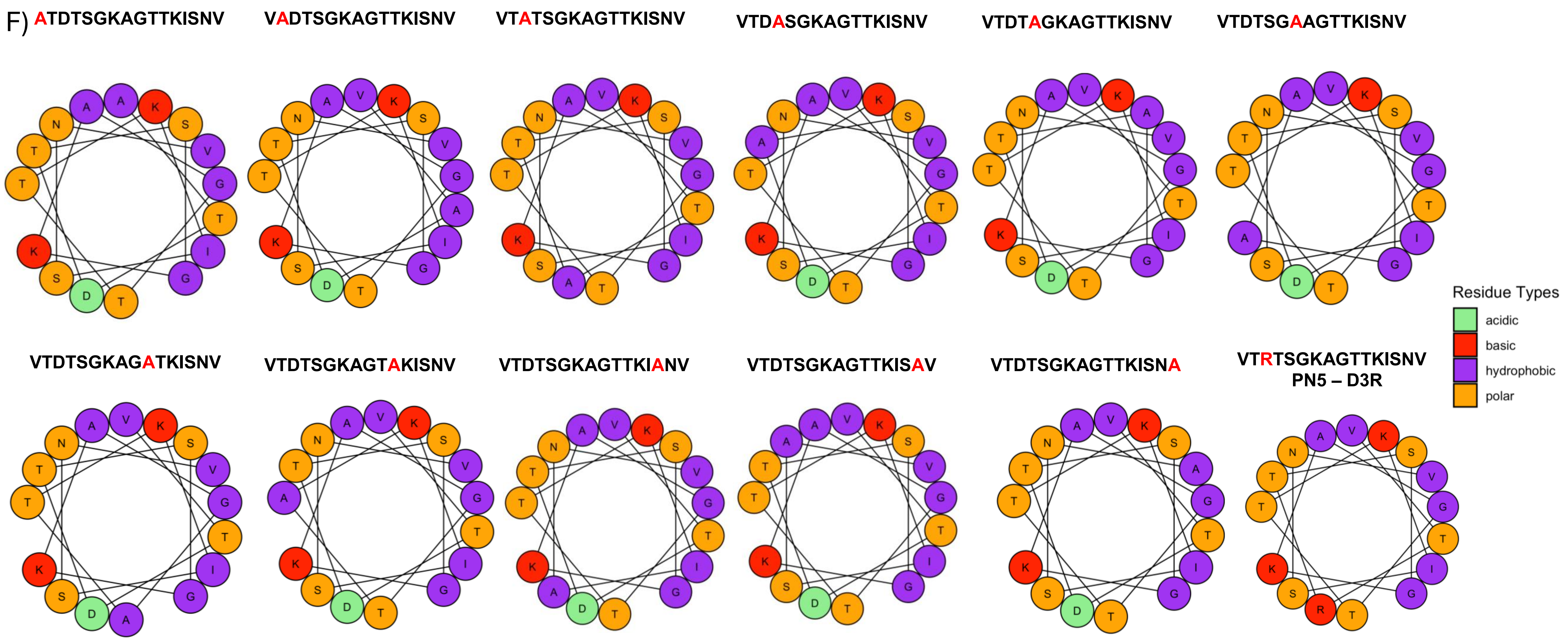

Helical wheel diagram of PN5-alanine and arginine substituted peptide analogs showing the relative position of different amino acids.
