# Supplementary material for: Antimicrobial peptides isolated from probiotics as an alternative to antibiotics against Salmonella infection
Source: Appl Environ Microbiol. 2026 Jan 30;92(2):e01654-25. doi: 10.1128/aem.01654-25 (PMC12915304; doi:10.1128/aem.01654-25)
Supplement: Supplemental legends — Descriptive captions for supplemental material. [file aem.01654-25-s0004.docx]

**SUPPLEMENTAL MATERIAL**

Supplementary Figure. 1: Antagonism between PN3 and PN5 against S. Typhimurium. 100 µl of ST (5 x10^6^ CFU/ml) was treated with different concentration of PN3 and PN5. When PN3 and PN5 were incubated together, a bacterial count of 8 log CFU/mL was observed at 15 mM. However, at 18 mM and 21 mM, a bacterial count of 6.3 log CFU/mL was observed, reducing the MIC of the PN5 from 21 mM to 18 mM. The Bacteria alone: NC, PN3: PN3 alone, PN5: PN5 alone and PN3+PN5: mixture of PN3 and PN5.

Supplementary Figure 2: **A and B** - 3D model of the PN3 and PN5 alanine and arginine substituted peptides analogs predicted by PEP-FOLD. **C and D** - Physiochemical properties of PN3 and PN5 alanine and arginine substituted peptide analogs, where AA=Amino acids, MW=Molecular weight, PI= Isoelectric point and GRAVY = Grand average of hydropathicity. **E and F** - Helical wheel diagram of PN3 and PN5 alanine and arginine substituted peptide analogs showing the relative position of different amino acids.

Supplementary Figure. 3: Growth curve showing the antimicrobial activity of arginine-substituted analogs of PN3 (A &B) and PN5 (C&D) peptides. Different concentrations of original peptide (15 mM-21 mM) and arginine analogs were added in a 96-well plate containing 100 µl of 5x10^6^ CFU/ml of ST, and the OD_600_ was measured every 30 mins for 12 hrs by Sunrise Tecan kinetic microplate reader.

Supplementary Table 1: *Salmonella* counts represented as log CFU/ml and the percentage of *Salmonella* inhibition in the MBEC assay.

Supplementary Table 2: Amino acid sequence of the original and arginine-substituted analogs of PN3 and PN5 peptides.

Supplementary Table 3: Details of *Salmonella* strains used in this study.
