# Supplementary material for: Antimicrobial peptides isolated from probiotics as an alternative to antibiotics against Salmonella infection
Source: Appl Environ Microbiol. 2026 Jan 30;92(2):e01654-25. doi: 10.1128/aem.01654-25 (PMC12915304; doi:10.1128/aem.01654-25)
Supplement: Supplemental tables — Tables S1 to S3. [file aem.01654-25-s0005.docx]

Supplementary Table 1: *Salmonella* counts represented as log CFU/ml and the percentage of *Salmonella* inhibition in the MBEC assay.

| **Groups** | **log (cfu/ml)** | **Percentage inhibition** |
| --- | --- | --- |
| PN3 | 0±0 | 100 |
| PN5 | 0±0 | 100 |
| PC | 0±0 | 100 |
| NC | 7.6±0.1 | 0 |

Supplementary Table 2: Amino acid sequence of the original and arginine substituted analogs of PN3 and PN5 peptides.

| **Original/substitution** | **Sequence** | **Net charge at pH 7** |
| --- | --- | --- |
| PN3 | VQAAQAGDTKPIEV | -1 |
| PN3-D8R | VQAAQAGRTKPIEV | 1 |
| PN3-E13R | VQAAQAGDTKPIRV | 1 |
| PN3-D8R&E13R | VQAAQAGRTKPIRV | 3 |
| PN5 | VTDTSGKAGTTKISNV | 1 |
| PN5-D3R | VTRTSGKAGTTKISNV | 3 |

| Bacterial spp. | Media | Culture conditions | Reference/source |
| --- | --- | --- | --- |
| *Salmonella* Albany | LB broth | 37 °C, aerobic, 12 h, 180 rpm | Laboratory collection |
| *Salmonella* Anatum | LB broth | 37 °C, aerobic, 12 h, 180 rpm | Laboratory collection |
| *Salmonella* Brenderup | LB broth | 37 °C, aerobic, 12 h, 180 rpm | Laboratory collection |
| *Salmonella* Enteritidis | LB broth | 37 °C, aerobic, 18-24 h, 180 rpm | Laboratory collection |
| *Salmonella* Heidelberg | LB broth | 37 °C, aerobic, 12 h, 180 rpm | Laboratory collection |
| *Salmonella* Javiana | LB broth | 37 °C, aerobic, 12 h, 180 rpm | Laboratory collection |
| *Salmonella* Muenchen | LB broth | 37 °C, aerobic, 12 h, 180 rpm | Laboratory collection |
| *Salmonella* Newport | LB broth | 37 °C, aerobic, 12 h, 180 rpm | Laboratory collection |
| *Salmonella* Saintpaul | LB broth | 37 °C, aerobic, 12 h, 180 rpm | Laboratory collection |
| *Salmonella* Typhimurium LT2 | LB broth | 37 °C, aerobic, 18-24 h, 180 rpm | John Gunn, OSU, Columbus |
| *Salmonella* Typhimurium Nalidixic acid resistant | LB broth/XLT-4 agar | 37 °C, aerobic, 18-24 h, 180 rpm | Laboratory collection |

Supplementary Table 3: Details of *Salmonella* strains used in this study.
